# Encapsulation of Caffeic Acid in Carob Bean Flour and Whey Protein-Based Nanofibers via Electrospinning

**DOI:** 10.3390/foods11131860

**Published:** 2022-06-23

**Authors:** Sema Zeren, Serpil Sahin, Gulum Sumnu

**Affiliations:** Department of Food Engineering, Middle East Technical University, 06800 Ankara, Turkey; sema.zeren@metu.edu.tr (S.Z.); gulum@metu.edu.tr (G.S.)

**Keywords:** active packaging, biodegradable, electrospun fiber

## Abstract

The purpose of this study was to introduce caffeic acid (CA) into electrospun nanofibers made of carob flour, whey protein concentrate (WPC), and polyethylene oxide (PEO). The effects of WPC concentration (1% and 3%) and CA additions (1% and 10%) on the characteristics of solutions and nanofibers were investigated. The viscosity and electrical conductivity of the solutions were examined to determine characteristics of solutions. Scanning electron microscopy (SEM), X-ray diffraction (XRD), thermogravimetric analyzer (TGA), differential scanning calorimetry (DSC), water vapor permeability (WVP), and Fourier transform infrared (FTIR) analysis were used to characterize the nanofibers. According to the SEM results, the inclusion of CA into nanofibers resulted in thinner nanofibers. All nanofibers exhibited uniform morphology. CA was efficiently loaded into nanofibers. When CA concentrations were 1% and 10%, loading efficiencies were 76.4% and 94%, respectively. Nanofibers containing 10% CA demonstrated 92.95% antioxidant activity. The results indicate that encapsulating CA into carob flour–WPC-based nanofibers via electrospinning is a suitable method for active packaging applications.

## 1. Introduction

Environmental factors such as pH, temperature, and light exposure, which can cause antioxidants to deteriorate, limit their bioavailability and bioaccessibility. Encapsulating antioxidants in biopolymer matrices is a successful approach to improve their bioavailability and stability [1]. Nano- and microtechnology allow antioxidants to be encapsulated in polymeric or nonpolymeric matrices [2]. Electrospinning has recently been proved to be a very viable method for encapsulating bioactive substances [1,2]. In general, this technique enables the polymer solution to flow through an electric field produced between the nozzle and receiver, resulting in the formation of nanofibers [3]. Using a high-voltage electrostatic field, a wide range of biopolymers, alone or in combination with other components, have been prepared. The electric field is required to overcome the surface tension of these polymer solutions and to stretch the steam ejected from a syringe, which is related to the Taylor cone’s instability. Concurrently, the jets partially or completely solidify due to solvent evaporation or cooling, and the fabrication of fibers into sheets or other shapes accelerates [4]. Electrospinning allows the production of fibers with ultra-fine structures, high porosity, a high surface-to-volume ratio, and customized morphology which can be employed in a variety of sectors including biological engineering, environmental protection, and within the energy and food packaging industry. It also has several advantages that make it suitable for producing active food packaging. Since it is a nonthermal process, it allows for the encapsulation of thermosensitive compounds. Furthermore, due to the rapid evaporation of solvents during operation, it reduces the amount of organic solvent remaining in the food system [5].

Traditional food packaging provides mechanical support as well as protection against environmental hazards such as micro-organisms, moisture, oxygen, odors, and dust. On the other hand, active food packaging is designed to scavenge undesirable substances or to release preserving agents such as antioxidants or antimicrobial agents [5]. There is currently a growing trend towards developing active food packaging materials from electrospun nanofibers. A wide range of synthetic and natural antioxidants have been investigated in order to develop active packaging and coatings. These active substances in the packaging films migrate to the food or absorb oxidative radicals from the food to improve the food’s quality and shelf-life. The antioxidants operate as a strong barrier against external microbial infections gaining access to the food surface, as well as destroy any oxidative stress areas that may exist in the food [6]. According to Shao et al. [7], pullulan-carboxymethylcellulose sodium nanofibers containing tea polyphenols were shown to significantly reduce weight loss, preserve firmness, and improve strawberry quality during storage. Curcumin-loaded electrospun nanofibers with very high encapsulation efficiency from zein and gelatin could be used as coatings for fatty food products, according to the findings of another study [8]. Additionally, grape-seed-extract-incorporated rye flour–WPC fibers and gallic acid-incorporated lentil flour fibers were promising examples of active electrospun nanofibers [9,10]

Caffeic acid (3,4-dihydroxycinnamic acid) is a phenylpropanoid and hydroxycinnamate metabolite found in plant tissues and food sources such as blueberries, apple cider, and coffee drink extracts. It is employed as a carcinogenic inhibitor which has antioxidant and antibacterial properties in vitro, hence aiding in the prevention of cardiovascular and atherosclerotic illnesses [11]. To the best of our knowledge, there is no study in the literature on encapsulation of caffeic acid into biodegradable nanofibers to be used as active packaging material.

Because of their good gas and liquid barrier properties, easy formability, availability, and low cost, petroleum-based synthetic films are used for food packaging. However, they are not biodegradable or sustainable, and they are also the source of significant disposal issues [12]. There is a high demand for eco-friendly packaging materials as a result of the environmental concerns. Biopolymers are a class of materials derived from naturally occurring and renewable resources. They have the potential to reduce our reliance on the extraction and processing of fossil fuels. Biopolymers are usually inexpensive, nontoxic, nutrient-rich, and edible. Biodegradable films can be made from lipids, polysaccharides, and proteins. In addition, flour can be a good alternative as it contains carbohydrates, protein, and fiber together. In the literature, there are studies in which different flour types, such as rye, rice, pea, lentil, and carob, are used to produce nanofibers via electrospinning [9,13,14,15,16].

Carob has a high fiber content (approximately 18%), mostly cellulose and hemicellulose, and also contains approximately 3–4% protein [17,18]. Cellulosic nanofibers are preferred due to their superior water vapor barrier properties with high mechanical strength [19]. Proteins are amphiphilic in nature because of their amino acid composition, and they provide many binding sites for bioactive substances, which are primarily governed by electrostatic attraction, hydrophobic interaction, hydrogen bonding, and covalent bonding. Proteins have several benefits over other materials, which has sparked a lot of interest in producing protein-based electrospun fibers [5]. Combining polysaccharides and proteins for food packaging can highlight the strengths of these two components while minimizing their limitations. There are studies in the literature that support this [9,20,21].

The objective of this study was to encapsulate caffeic acid into biodegradable nanofibers made of carob flour, to be used as active packaging material. Since the protein ratio in carob is low, the addition of WPC was assumed to be more suitable for developing packaging materials in this study. A carob flour–whey protein concentrate blend has never been used as a nanofiber material before. The crystalline structure of the cellulose and the complex secondary and tertiary structures of the proteins make them impractical for producing electrospun nanofibers due to insufficient entanglement. Several methods, such as denaturation, the use of an appropriate solvent, and blending with other polymers, can be used to ensure the successful electrospinning of globular proteins [5,19]. In this study, microwave treatment was selected as the heat treatment to increase spinnability of biopolymers. Additionally, microwave heating was shown to be a good method for providing bead-free homogeneous nanofibers because internal heating generates higher internal pressure, which promotes the release of free amino groups and increase in viscosity [9]. Studies related to the microwave-pretreated active electrospun nanofibers were very limited. Additionally, adding water-soluble and biodegradable thermoplastic polymer polyethylene oxide (PEO) to the whey protein isolate solution was shown to increase the solution’s spinnability due to PEO chain entanglement with biopolymer molecules and also the charge-counteracting effect of PEO on biopolymers [22,23]. Thus, PEO was added into carob flour–WPC blend in this study to obtain bead free and homogenous nanofibers.

## 2. Materials and Methods

### 2.1. Materials

Carob flour was purchased from Havancızade Gıda Gıda Co., Inc. (İstanbul, Turkey). Whey protein concentrate (80% protein on a dry-weight basis) was provided from Proteinocean Gıda Co. Inc. (Ankara, Turkey). Caffeic acid (CAS #: 331-39-5), polyethylene oxide (900 kDa molecular weight, CAS #: 25322-68-3), and sodium hydroxide pellets (CAS number: 1310-73-2) were obtained from Sigma Aldrich Chemical Co., (St. Louis, MO, USA). Polyoxyethylene sorbitan monooleate (Tween80) (density: 1.064 g/m^3^, viscosity: 400–620 cps at 25 °C, CAS #: 9005-65-6) was supplied from Merck (Darmstadt, Germany).

### 2.2. Solution Preparation

PEO was dissolved in the distilled water with magnetic stirrer (MaxTir 500, Daihan Scientific Co, KR) for 24 h at 400 rpm to obtain 2.5% (*w*/*v*) homogenized solution. Then, carob flour (3% *w*/*v*) and whey protein concentrate (1%, and 3% *w*/*v*) were added to the solution at different concentrations (Table 1).

Solutions were mixed with high-speed homogenizer (IKA T25 Digital Ultra-Turrax; IKA^®^-Werke GmbH&Co. KG, Staufen, Germany) at 10,000 rpm for 3.5 min. pH of the solutions was adjusted to 10 by adding 8 M NaOH. pH of the solutions was measured by pH Portable Meter (SG2 SevenGoTM, Mettler, Toledo, OH, USA). Temperature of the solutions was brought to 80 °C by microwave heating (450 W for 2.5 min) (Advantium Oven TM, General Electric Company, Louisville, ABD). At the same time, caffeic acid (CA) was dissolved in 80% aqueous ethanol by magnetic stirrer at 750 rpm for an hour. Then, it was incorporated into the electrospinning solutions to obtain 1% and 10% (w/w, % in solid fibers) of CA content in solid fibers. Tween80 (2% *w*/*v*) was added to the mixtures as surfactant. Then, final solution was stirred further by using magnetic stirrer for 30 min at 750 rpm for complete homogenization.

### 2.3. Solution Properties

#### 2.3.1. Rheological Properties

Rheological properties of the solutions were analyzed by a controlled strain rheometer (Kinexus, Pro+ Rheometer, Malvern, UK) with a titanium cone and plate geometry (4° cone, 40 mm diameter, and 1 µm gap) at 25 °C for shear rates varying between 0.1 s^−1^ and 100 s^−1^. Shear rate versus shear stress values were recorded. Power law model was chosen to determine the flow behavior index (n) and the consistency index (k). Measurements were duplicated.

#### 2.3.2. Electrical Conductivity

Solution’s electrical conductivity was measured by using conductivity meter (InoLab^®^ Cond 7110, Wissenschaftlich-Technische Werkstätten GmbH, Wheilheim, Germany) at room temperature. Measurements were conducted twice.

### 2.4. Electrospinning Process

To obtain films made of nanofibers, the electrospinning process was applied. Each solution was put into 5 mL syringe with an inner diameter of 11.53 mm and a 21-gauge steel needle. It was placed horizontally into the electrospinning equipment, which includes a high-voltage source, a syringe pump, and a rectangular metal collector (Nanoweb 103, Mersin, Turkey). The positively charged electrode was linked to the needle while the negatively charged electrode was linked to a metal collector wrapped with aluminum foil. The distance between the collector and the needle’s tip was fixed at 0.3 m. Experiments were carried out at room temperature with a relative humidity of 30–40%, a flow rate of 0.8 mL/h, and a voltage of 12 kV.

### 2.5. Characterization of Films

#### 2.5.1. Morphological Analysis

Samples’ morphology was analyzed by using scanning electron microscopy (SEM) (Nova NanoSEM 430, Hillsboro, OR, USA). Samples were coated with gold palladium and scanned at 10,000× magnification level. Diameters of nanofibers were measured from the SEM images by using Image J analysis software V 1.50i (Bethesda, MD, USA). Then, average fiber diameter was calculated from one hundred randomly selected nanofibers for each sample.

#### 2.5.2. Differential Scanning Calorimetry

The thermal analysis of the electrospun nanofibers was performed using a differential scanning calorimeter (Pyris 6 DSC, PerkinElmer, Waltham, MA, USA). Approximately 5 mg of sample was put into a hermetically sealed aluminum pan. As a reference, an empty pan was used. After cooling to –60 °C, each pan was heated to 250 °C at a rate of 10 °C/min. The glass transition temperature, melting temperature, and melting enthalpy of each sample were determined using differential scanning calorimetry (DSC) thermograms. The DSC measurements were carried out in duplicates [15].

#### 2.5.3. Thermogravimetric Analysis

Thermogravimetric analysis (TGA) was conducted to examine the weight change of the fibers, caffeic acid, whey protein, carob flour, and PEO as a function of temperature by thermo-gravimetric analyzer (Perkin Elmer Pyris 1). About 5 mg of sample in powder form was heated from room temperature to 500 °C at a rate of 10 °C/min with nitrogen [24].

#### 2.5.4. X-ray Diffraction

The X-ray diffractometry (XRD) data of the nanofibers were obtained using Ultima IV X-ray diffractometer (Rikagu, Japan) with Cu Kα radiation in a range of 2θ = 5–70° [25].

#### 2.5.5. Fourier Transform Infrared Analysis

Nanofibers were analyzed using a Fourier transform infrared (FTIR) spectrometer (IR-Affinity1, Shimadzu Corporation, Kyoto, Japan) with an attenuated total reflectance (ATR) attachment. Data were collected at a resolution of 2 cm^−1^ in the wavenumber range of 4000–600 cm^−1^ [26].

#### 2.5.6. Water Vapor Permeability

Modified version of ASTM E-96 method was used to determine water vapor permeability (WVP) [9]. Thickness of each film was measured before the experiment by using digital micrometer (LYK 5202, Loyka, Ankara, Turkey). Cylindrical test cups with a 0.04 m diameter were filled with 30 mL of distilled water. Films were placed in between the cup and the ring cover of each cup coated with sealant and held with screws around the cup. Then, cups were placed into the desiccator filled by silica gels. Films were assumed to be subjected to 100% RH from the inside. Test cup was weighted initially, and then weight changes of the cups were recorded over 12 h period at 1 h intervals. Measurements were replicated twice. During the measurement, relative humidity (RH) and temperature inside the desiccator were recorded using a digital hydrometer (ThermoPro TP50, Atlanta, GA, USA). From the weight loss versus time graph, water vapor transmission rate (WVTR) was calculated, and WVP was found using the equation below:

(1) $$\mathrm{WVP}=\frac{\mathrm{WVTR}\times \Delta X}{P_{\mathrm{wi}}-P_{\mathrm{wo}}}\;$$

where

WVP: water vapor permeability (g·m^−1^ ·s^−1^ ·Pa^−1^);
ΔX: film thickness (m);
WVTR: water vapor transmission rate (g·m^−2^·s^−1^);
P_wi_: partial pressure of the water vapor inside the cup (Pa);
P_wo_: partial pressure of the water vapor outside the cup (Pa).

#### 2.5.7. Antioxidant Activity

A modified version of the 2,2-diphenyl-1-picrylhydrazyl (DPPH) technique was used to determine the antioxidant activity of nanofibers containing caffeic acid [27]. A total of 10 mg nanofiber sample was combined with 25 mL of 80% (*v*/*v*) ethanol–water solution and left to dissolve for 2 h before centrifuging at 10,000 rpm for 3 min. An amount of 0.5 mL was added to 3.5 mL of 0.6 mM DPPH solution and incubated for 1 h in the dark. A spectrophotometer was used to measure the absorbance at 517 nm (UV 2450, Shimadzu, Columbia, MD, USA). Control was the mixture of 0.5 mL of 80% (*v*/*v*) ethanol–water solution and 3.5 mL of 0.6 mM DPPH solution. Methanol was used as blank. The measurements were taken twice. Using the formula below, the antioxidant activity (AA%) of the fibers was calculated.

(2) $$\mathrm{AA}\left(\%\right)=\frac{A_{\mathrm{control}}-A_{\mathrm{sample}}}{A_{\mathrm{control}}}\times 100$$

where

AA: antioxidant activity of fibers;
A_control_: absorbance of the control sample at 517 nm;
A_sample_: absorbance of the fibers at 517 nm.

#### 2.5.8. Loading Efficiency

The method described in Aydogdu et al. [28] was used to determine the caffeic acid loading efficiency in the nanofibers. A spectrophotometer was used to capture the spectrum of caffeic acid dissolved in 80% ethanol–water solution at 300–600 nm, with maximal absorption at 325 nm. By initially dissolving 10 mg of electrospun fibers in 25 ml of 80% ethanol–water solutions, the loading efficiency of caffeic acid in the electrospun fibers was measured. The absorbance values of solutions at wavelengths of 325 nm were determined using a spectrophotometer after appropriate dilutions were carried out (UV2450, Shamadzu, Columbia, MD, USA). A preset caffeic acid standard calibration curve was used to determine the quantity of caffeic acid in the fibers. Using the formula below, caffeic acid’s loading efficiency (LE%) was calculated.

(3) $$\mathrm{LE}\left(\%\right)=\frac{\left(\mathrm{Calculated}\;\mathrm{caffeic}\;\mathrm{acid}\;\mathrm{amount}\right)}{\mathrm{Theoritical}\;\mathrm{caffeic}\;\mathrm{acid}\;\mathrm{amount}}\times 100$$

where LE: loading efficiency of the fibers.

#### 2.5.9. Biodegradability

The biodegradability of films was assessed with some modification as reported by da Silva Filipini et al. [29]. Weight fluctuations would be inaccurate due to remaining soil on film surfaces; hence, they were evaluated qualitatively. Soil was poured into a cylindrical plastic cup with a diameter of 20 cm and a height of 15 cm. A 2 cm × 3 cm sample was placed inside a support and buried in the soil at a depth of 10 cm. The plastic cup was kept at room temperature (21 ± 2 °C) and relative humidity (65 ± 5%). To keep the compost moist, water was sprayed once a day. Samples were carefully taken out every five days until complete degradation and recorded by a camera.

### 2.6. Statistical Analysis

Minitab software was used to conduct statistical analysis (Minitab Inc., State College, PA, USA). ANOVA was used to determine whether there were any significant differences between treatments. Tukey’s multiple comparison test was used to determine whether there were significant differences in the data (*p* ≤ 0.05).

## 3. Results and Discussion

### 3.1. Solution Properties and Their Relation to Fiber Morphology

The diameter, size, and morphology of the electrospun nanofibers are all controlled by a variety of parameters, including solution properties, process conditions, and ambient parameters. By adjusting these parameters to meet the needs of the application, various polymers can be electrospun into nanofibers with the desired fiber diameter and morphologies [19]. The effect of solutions’ properties, which are viscosity and electrical conductivity, was investigated in this study by maintaining constant ambient conditions and process parameters. The viscosity of a solution is determined by the interactions of its constituent molecules and is dependent on the concentrations and properties of the solutes and reagents utilized, and also on the pH [30]. Electrical conductivity facilitates the elongation of droplets and the production of a single or many jets [31]. A solution with low conductivity produces fibers with larger diameters, whereas a solution with high conductivity can produce excessively small fibers such as spider-net fibers. As a result of the solution’s efficient conductivity, the electrostatic interaction on the jet increases, and simultaneous effective jet elongation results in fibers with the smallest diameter. Thus, conductivity can help to achieve the desired fiber diameter and morphology [32]. In Table 2, consistency index (k), flow behavior index (n), the electrical conductivity of the solutions, and the average diameters of electrospun fibers obtained from solutions with different WPC and CA concentrations can be seen.

**Figure 1:**
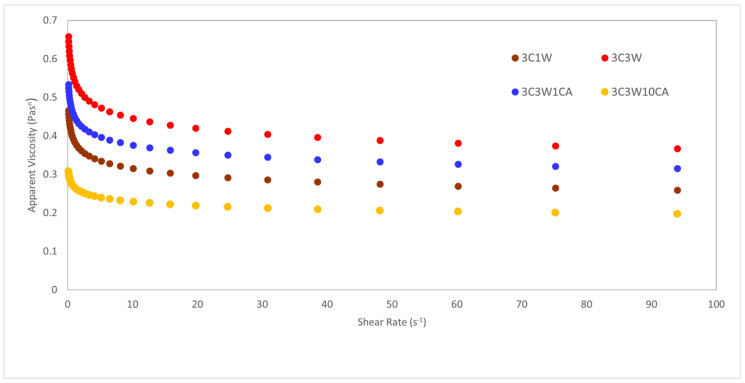
Apparent viscosity of the solutions with respect to shear rate.

Figure 1 shows the apparent viscosity versus shear rate graph of the solutions. The power law model was followed by all electrospinning solutions, which had high coefficients of determination values (r^2^ = 0.998–0.999).

**Figure 2:**
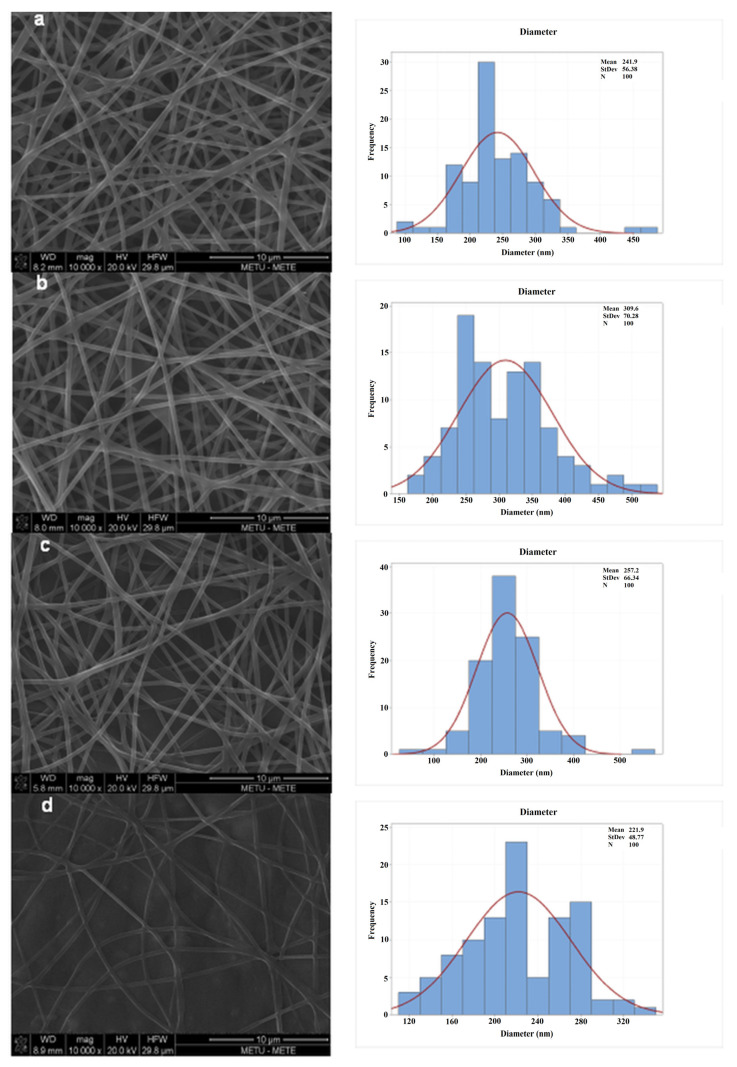
SEM images and fiber diameter distributions of the nanofibers: (**a**) 3C1W, (**b**) 3C3W, (**c**) 3C3W1CA, (**d**) and 3C3W10CA.

Shear-thinning behavior was observed since the apparent viscosity of the solutions decreased as the shear rate increased. Flow behavior index values (n), which were less than 1, also validated that the solutions had shear-thinning properties. Shear-thinning behavior has also been observed in other electrospinning studies [9,27,33,34]. The highest consistency index value was found in the solution with the highest whey protein concentration containing no caffeic acid. Additionally, k increased from 0.386 to 0.545 when the WPC concentration increased. Similarly, in the previous studies it was found that addition of whey protein increased the viscosity of the electrospinning solution, resulting in the formation of continuous bead-free nanofibers. In these studies, it was discovered that by supporting the whey protein with easily spinnable polymers such as PEO and performing other treatments such as drastically changing the pH of the solutions from their isoelectric point or applying heat treatment, the protein unfolding and amino group release are accelerated, and the molecular entanglement in the polymer solution is increased [9,23,35,36,37]. The same trend was observed in apparent viscosity. By increasing the WPC concentration from 1% to 3%, the apparent viscosities of the solutions increased (Figure 1). The apparent viscosity of polymer solutions is a function of their concentration and molecular weight. This helps us to explain why apparent viscosity increases as protein content increases [21]. Furthermore, there was a strong positive correlation between consistency index and average fiber diameter (r = 0.948, *p* = 0.05). Average fiber diameters of the films ranged between 222 nm and 310 nm. The sample of 3C3W presented the highest average fiber diameter (310 ± 70 nm). Higher k values in solutions resulted in nanofibers with higher diameters as the number of molecular entanglements in the solution rose [28]. In addition, when the amount of caffeic acid in the solution was increased, the k values decreased from 0.451 to 0.267. Caffeic acid was dissolved in the ethanol–water solution then added to carob–WPC–PEO solutions, so that when the amount of caffeic acid in the solution increased, so did the amount of ethanol–water in the solution. As a result, the solution became less viscous, and the k value reduced significantly. Similarly, gallic-acid-enriched electrospun nanofibers showed a lower viscosity than those without the gallic acid [28]. Figure 2 shows the SEM images and the diameter distributions of the nanofibers. According to the SEM images, bead-free, homogenous nanofibers were obtained from the different carob flour–WPC combinations.

The addition of WPC reduced the sticky appearance of the nanofibers. In addition, as shown in Figure 2, encapsulating caffeic acid into the nanofibers did not destroy the nanofiber structure, and continuous bead-free nanofibers were obtained.

As can be seen in Table 2, an increase in the whey protein concentration did not affect the electrical conductivity of the solution significantly. However, the addition of caffeic acid and increase in the caffeic acid content increased the conductivity significantly (*p* < 0.05). After the addition of caffeic acid to the solution, the pH of the solution was adjusted again to alkaline conditions with the addition of NaOH. Alkaline solutions contain many Na^+^ and OH^−^ ions, thus causing an increase in the conductivity [23]. When conductivity increased, there was a decrease in the average fiber diameter. Similar to the study of [28], the nanofiber sample with the smallest average diameter was obtained from the solutions having the highest conductivity. As a result, it is reasonable to state that decreasing a solution’s viscosity while increasing solution conductivity promotes the formation of smooth fibers with smaller diameters, increasing the solution’s elongation capacity [20]. The same trend was observed in polylactic acid–tea polyphenol nanofibers, in which as the tea polyphenol concentration increased, fiber diameter decreased from 753 nm to 493 nm. As the TP content of the spinning solution increased, so did the solution’s conductivity, which resulted in increased electrostatic repulsion between ejection flows and the formation of thinner ejection flows; eventually, smaller-diameter fibers were collected [30].

### 3.2. Differential Scanning Calorimetry

Table 3 shows the glass transition temperature (T_g_), the melting temperature (T_m_), and enthalpy change (ΔH_m_) of the nanofibers.

The glass transition temperature (T_g_) is related to the softening point from the glassy state to rubbery state. T_g_ values of nanofibers decreased significantly with an increase in WPC concentration. Additionally, in the study of Ignatova et al. [38], due to the increase in the hydrogen bond formation between the water molecules and hydroxyl residues on the polymer chains, which had a plasticization effect, T_g_ decreased. Having both T_m_ and T_g_ values indicates that nanofibers had a semi-crystalline structure showing both amorphous and crystalline properties. T_m_ indicates the melting point of the crystalline phase. The melting temperature of the nanofibers varied between 60.9 and 63.9 °C, which was lower than melting temperature of pure PEO. The T_m_ of PEO was reported as 71.5 °C [15]. Similar results were also seen in the different studies [9,10,15,24,27]. One of the reasons for the depression in the melting temperature and the enthalpy might be related to the interaction between PEO and other polymers, carob flour, and whey protein. This interaction might have disrupted the crystallinity of PEO. Moreover, the decrease in T_m_ was higher when the whey protein concentration increased, supporting this reason. The other reason might be related to the working principle of electrospinning. During electrospinning, the rapid solidification process of the stretched chains causes the polymer chains to remain in a mostly noncrystalline state [27]. The melting point of the crystal caffeic acid was recorded as 203 °C [38]. No peak corresponding to that temperature was found in the DSC thermograms of the nanofibers containing 1% and 10% caffeic acid, showing that CA was encapsulated in the nanofibers successfully.

### 3.3. Thermogravimetric Analysis

**Figure 3:**
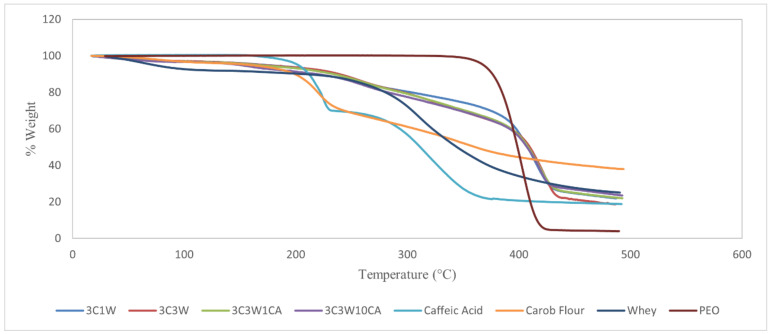
Thermogravimetric curves of the electrospun nanofibers, carob flour, whey, PEO, and caffeic acid.

TGA is a method of determining the thermal stability of materials by measuring the weight change as a function of temperature. Figure 3 depicts the weight loss curve of polymers and fibers against temperature.

PEO had one degradation stage with a beginning temperature of roughly 350 °C, and at almost 410 °C, nearly 95% of the sample weight degraded. Carob flour showed two stages of degradation at around 220 °C and 350 °C. The T_onset_ of whey protein was nearly 270 °C, and at the end of the experiment nearly 20% of the sample weight remained. Caffeic acid, which was stable up to 150 °C, showed two thermal degradation steps at nearly 230 °C and 325 °C, respectively. The first stage of weight loss combines melting and degradation of CA while the second decomposition step might be linked to acid decarboxylation [11]. The nanofibers had a two-stage degradation profile, but the TGA of all nanofibers had a slight initial weight loss up to 100 °C due to the evaporation of solvents, mostly free water. The first degradation took place between 200 and 300 °C, which might be linked to polysaccharide and whey protein degradation, while the second degradation took place between 400 and 450 °C, which could be linked to PEO degradation. The same trend has also been observed in rye-flour-based nanofibers [9]. Furthermore, the nanofibers’ 80 % weight degradation temperature was 50 °C greater than the temperature of pure powdered PEO. These variations could be explained by the amounts of these compounds in the fibers, as well as their varying abilities to form hydrogen bonds, which improve heat stability, as found by Colín-Orozco et al. [22]. The first degradation curve of the caffeic-acid-enriched nanofibers was similar to that of 3C3W, while the second one had a lower slope. This finding suggests that the caffeic acid addition slowed down the heat degradation, implying a strong influence on intermolecular interactions as well as its ability to scavenge radicals generated during thermal degradation [27]. Furthermore, whereas caffeic acid degraded at 325 °C, active nanofibers showed no additional weight loss at that temperature, indicating the successful encapsulation and thermally stabilized caffeic acid components. Caffeic acid has also been observed to improve thermal properties of nanofibers in other studies. Main degradation temperatures of the ethylene vinyl alcohol copolymer and caffeic-acid-based films increased by about 25 °C [11]. Additionally, caffeic acid addition to polypropylene films showed improved thermal resistance compared to the addition of flavanones, chlorogenic, and trans-ferulic acids. This was explained by the enhanced antioxidant property due to additional resonance with the presence of a second hydroxyl group in the ortho- or para-position. Because of the high density of conjugated structures, they are particularly effective at scavenging free radicals and thereby slow down the thermo-oxidative degradation process [39].

### 3.4. X-ray Diffraction

**Figure 4:**
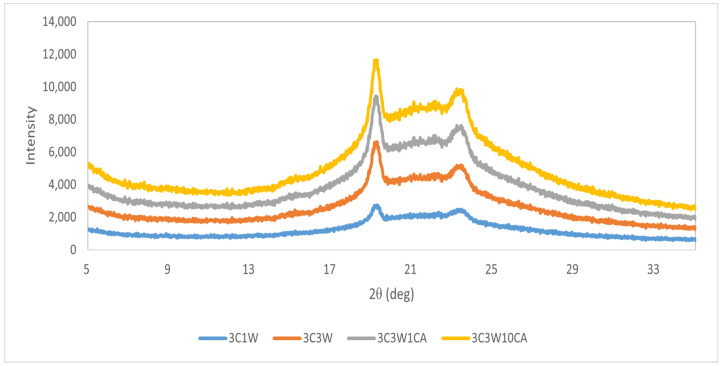
X-ray diffractogram of electrospun nanofibers.

All four samples had a similar diffraction pattern with two highly reflected peaks, according to the XRD results presented in Figure 4. Thus, the composite nanofiber samples are semi-crystalline materials with both amorphous and crystalline structures exhibiting broad peaks of low intensity.

At 2θ = 19° and 2θ = 23°, all samples displayed distinct peaks. The crystallinity of PEO could be responsible for those peaks. XRD examination of pure PEO revealed peaks at 19.03° and 23.20° in previous research [24]. Similar peaks could account for the homogeneous distribution of carob flour, whey protein, and caffeic acid through PEO. They did not appear to have any unusual crystal formations. This also demonstrated how materials interacted powerfully [15]. As seen in DSC results (Table 3), XRD results also show that the interaction of PEO with other polymers in the solution, increasing the whey protein concentration, or the nature of the electrospinning might have resulted in lower crystallinity than PEO. Another reason could be the rapid solvent evaporation during electrospinning as explained in the study of Ignatova et al. [40]. The rapid solvent evaporation may also be responsible for the amorphous state of caffeic acid phenethyl ester in electrospun fiber mats, resulting in inadequate drying time for the molecular organization required to form a crystal lattice [40].

### 3.5. Fourier Transform Infrared Analysis

**Figure 5:**
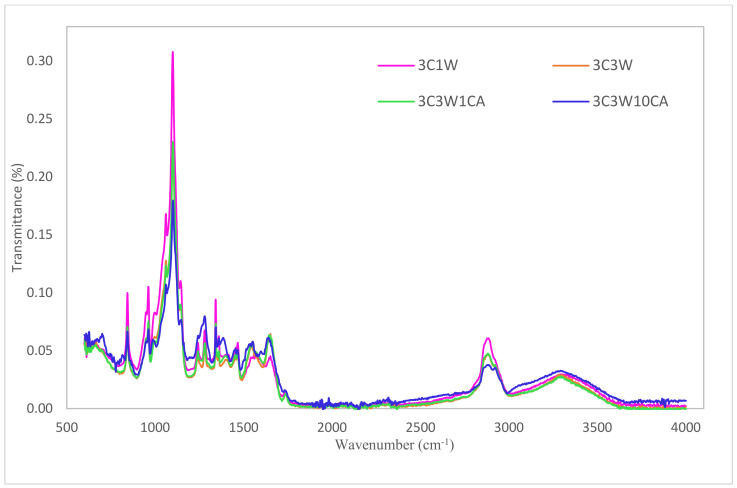
FTIR spectra of nanofibers.

Fourier transform infrared spectroscopy (FTIR) was used to detect the interactions and linkages in the structure of nanofibers. FTIR spectra of the nanofibers are shown in Figure 5.

The presence of the PEO was confirmed by the peaks at about 1100 cm^−1^ and 2850 cm^−1^. Peaks at roughly 2850 cm^−1^ belong to the methylene group molecular stretching, whereas peaks at 1100 cm^−1^ correspond to the combination of the ether and stretching methylene groups in PEO, as indicated in the previous studies [35,41]. Peaks in the 1500–1700 cm^−1^ region and also the increase in the peak intensity parallel to the WPC concentration indicated the presence of WPC. The nanofibers’ spectra revealed a distinctive peak at about 1650 cm^−1^, which was linked to the amide-I region and found in proteins. The amide-II band, which occurs predominantly due to N=H in-plane bending vibrations, is ascribed to the band that appears at around 1540 cm^−1^. This peak was also visible in the spectrum of pure whey protein powder [9,35]. As was explained in Uygun et al. (2020), who also worked with carob flour, the peaks located around 960 cm^−1^ belonged to the section of starch, amylose, and amylopectin monomer glucose units which was found in the carob flour. Peaks near 960 cm^−1^ in the spectra of nanofibers were attributed to vibrations caused by C-O-C glycosidic linkages. The resulting composite fibers retained all the characteristic bands associated with PEO, carob flour, and WPC. However, minor differences in the spectra of caffeic-acid-encapsulated nanofiber were discovered. Similar to another study revealing the spectrum of caffeic acid, peaks at 1650 cm^−1^ due to the carboxyl group (C=O), and peaks at 1614 cm^−1^, 1608 cm^−1^, and 1456 cm^−1^ coming from the aromatic ring (C=C), were observed [42]. The shifting of the bands, such as the amide-III peak shifting from 1298 cm^−1^ (3C3W) to 1276 cm^−1^ (3C3W10CA), and also changes in the peak intensities, for example, methylene stretching at 2850 cm^−1^, were less intense for caffeic-acid-enriched films than the control film, and could be attributed to an interaction between caffeic acid and the polymers in the nanofiber’s composition. The shift in the amide-III was also observed in the caffeic-acid-containing starch–chitosan film. It was suggested that this might be due to the electrostatic interaction between the charged amino group (NH^+3^) of chitosan (or gelatin) and the charged carboxylic group (COO^−^) of antioxidants’ phenolic ring (ferulic acid and caffeic acid) [43]. These findings show that caffeic acid was successfully incorporated into electrospun nanofibers.

### 3.6. Water Vapor Permeability

One of the most essential features in food packaging applications is water vapor permeability (WVP). Food packaging materials with adequate barrier qualities can improve packaging conditions by reducing moisture transfer between the food product and the environment [44]. The WVP values of the electrospun nanofibers can be seen in Table 4, which varied between 2.95–1.38 × 10^−10^ g·m^−1^ ·s^−1^ ·Pa^−1^.

The comparison of WVP values between different samples revealed the effect of whey protein concentration variation in nanofibers and antioxidant incorporation into nanofibers. In another study, the WVP of a carob–rice starch–PEO blend of electrospun fibers ranged between 2.73 and 1.68 × 10^−12^ g·m^−1^·s^−1^ ·Pa^−1^ [15]. The WVP difference between the studies could be explained by the composition and concentration difference of the solutions, since the PEO concentration was higher, and WPC was used instead of rice starch in our study. The hydrophilic nature of both WPC and PEO contributed to the higher water vapor permeability. According to our result, while nanofibers with 3% WPC have the highest barrier property against water vapor, 1% WPC has the lowest barrier property. The diameter of the nanofibers and the water vapor barrier characteristic have a positive relationship [45]. Nanofibers with a high polymer content had a significantly larger diameter and lower WVP values. Similarly, it was observed that WVP values of the nanofibers decreased as the total polymer content of the solutions increased. The reason behind that might be explained by the increased number of fiber junctions because the porosity of electrospun mat decreased as the fiber diameter increased. The porosity of nanofibers has been shown to have an effect on the WVP values. Additionally, as the polymer concentration increased, the viscosity increased, reducing the mobility of molecules. Thus, fibers with a high total polymer content exhibited a high viscosity and low water vapor transfer [45]. In another study, addition of lime peel extract to the lime peel pectin films lowered the WVP. It is explained by the film compositions (hydrophilic or hydrophobic in nature), and concentration may cause the different behavior of film WVP values due to the intermolecular interaction between the film matrix and the extract, including the tortuosity pathway of water molecules [46]. Although a change in the caffeic acid concentration did not significantly affect the WVP, addition of the caffeic acid led to a significant increase in the WVP. As was explained in the previous sections, although the hydrophobic property of phenolic acids disrupts water vapor transport through the films, addition of ethanol causes decreases in solution viscosity and fiber diameter. Decreases in the fiber diameter might have an influence on increases in the WVP. An increase in the caffeic acid concentration led to a slight decrease in the WVP. In the study of Araghi et al. [44], caffeic acid addition to the fish gelatin had a positive effect on permeability. However, the concentration changes in ferulic acid had no effect on the water vapor permeability of fish gelatin. It could be due to the significant number of hydroxyl groups in ferulic acid that can bind with water. In another study, the addition of unoxidized phenolic acids (ferulic, caffeic, and gallic acid) had no significant effect on the WVP of soy protein film [47].

### 3.7. Loading Efficiency (LE%) and Antioxidant Activity (AA%)

The efficiency of loading the caffeic acid into the nanofibers and the antioxidant activity of the nanofibers are given in Table 5.

The loading efficiencies were 76.4% and 94.0% for 1% and 10% CA concentrations, respectively. It can be concluded that electrospinning is a very efficient process for encapsulating sensitive bioactive compounds since it is performed at room temperature. Nanofibers containing 10% caffeic acid had an extremely high encapsulation efficiency. It was higher than those previously reported for other encapsulation technologies. In the study by Fathi et al. [48], the encapsulation efficiency of the caffeic-acid-loaded solid lipid nanoparticles was 71.21%. Encapsulation of caffeic acid phenethyl ester in skim milk microcapsules via spray-drying reached 41.7% encapsulation efficiency [49]. Caffeic acid (3,4-dihydroxycinnamic acid) found in various agricultural foods functions as an antioxidant by scavenging oxygen-free radicals and chelating pro-oxidant metal ions [48]. Due to increased resonance stabilization and o-quinone or p-quinone production, the presence of a second hydroxyl group in the ortho- or para-position is known to boost antioxidative activity. This helps to explain why caffeic acid and its phenethyl ester have such great antioxidative activity [50]. 3C3W had 0.85% antioxidant activity, which came from the phenolic compounds already found in the carob flour. Additionally, previous studies proved that carob flour has bioactive substances [18,51]. As the caffeic acid concentration increased, so did their antioxidant activity, which is also indicated by the effective electrospinning encapsulation.

### 3.8. Biodegradability

**Figure 6:**
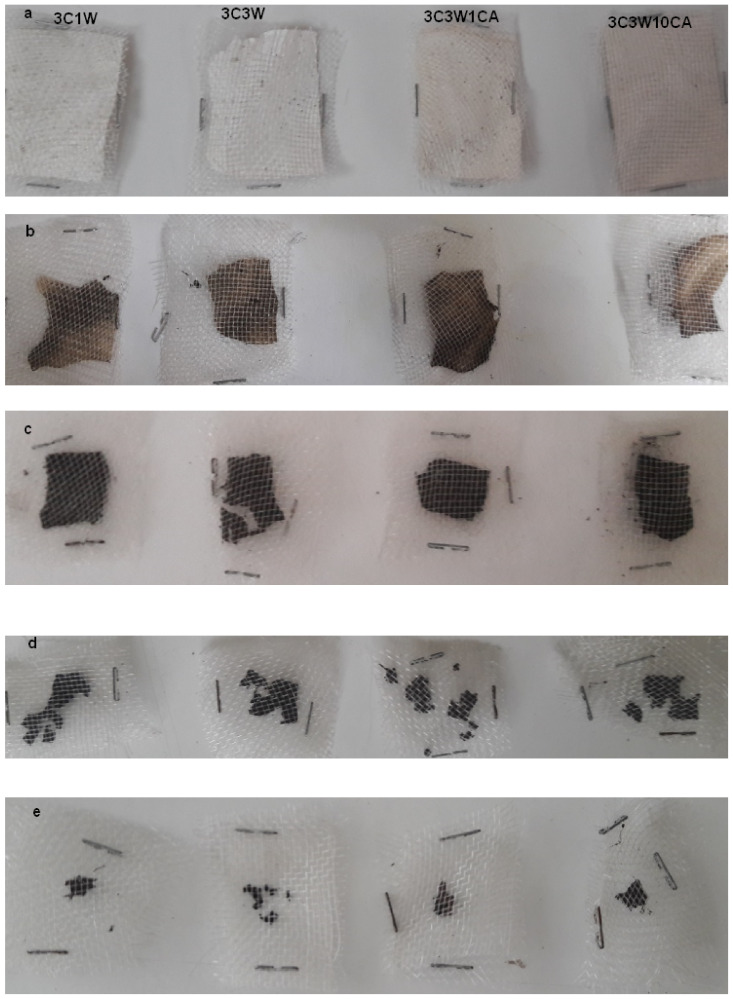
Biodegradability of films at the (**a**) beginning, (**b**) 5th day, (**c**) 10th day, (**d**) 15th day, (**e**) and 20th day; order of the samples in each row: 3C1W, 3C3W, 3C3W1CA, and 3C3W10CA.

Biodegradation is the biochemical material conversion process in water, biomass, carbon dioxide, or methane. The biodegradation of the polymer is divided into two stages. First, the process of reducing polymer chain carbon bond breakage in terms of heat, humidity, and the presence of microorganisms occurs. Second, when shorter chains become energy sources for microorganisms which are bacteria, fungi, or algae, part of the biodegradation process begins. Complete biodegradation is achieved when carbon compounds are converted into water, biomass, or carbon dioxide by micro-organisms [52]. Biodegradation of the films in the soil was observed visually (Figure 6).

The reduction in the area of the biodegraded films was observed every five days until the degradation was complete. The first sign of deterioration in the films was a shift in color. At the end of the fifth day, the color of the films had darkened, and the films had shrunk. In the following days, the films’ original appearance and structural integrity had been deteriorated, revealing rougher, degraded surfaces with porous pits and holes. After 15 days, the films were significantly disintegrated. At the end of the 20th day, the areas of the samples were significantly reduced. da Silva Filipini et al. [29] reported that because of their sensitivity to water, films with high solubility tend to biodegrade quickly because the components of the film structure are available for micro-organisms to digest. Additionally, Alqahtani et al. [53] stated that the weight loss could be a result of the soil microflora disintegrating or the film components becoming soluble due to the addition of water to the soil. In the literature, cassava starch films with natural extracts (green tea and basil) [54] and with yerba mate extract [55] showed a degradation time of 12 days, and methylcellulose and jambolao (Syzygium cumini) skins extract films showed a degradation time of 15 days [29]. All the films produced in the present study are biodegradable. This is a promising result for environmentally friendly applications.

## 4. Conclusions

Caffeic acid was successfully encapsulated in carob flour and whey-protein-based electrospun nanofibers in this study. The increase in the WPC concentration led to an increase in the fiber diameter; thus, water vapor barrier properties were improved from 2.95 × 10^−10^ to 1.38 × 10^−10^ g·s^−1^ ·m^−1^ ·Pa^−1^. CA inclusion in carob–WPC–PEO electrospun nanofibers was validated morphologically, chemically, and thermally. When the concentration of CA was increased, the diameter of the fibers decreased. CA nanofibers synthesized in this study exhibited a uniform and bead-free structure. The FTIR spectrum confirmed that there was a molecular interaction between CA and carob–WPC–PEO. Nanofibers containing 10% CA with 94% loading efficiency have shown that electrospinning is an effective approach to encapsulating bioactive chemicals into biomaterials. They also had 92.95% antioxidant activity, proving that CA is a suitable material for active packaging because of its antioxidant characteristics. TGA and DSC results also support the loading efficiency findings. Finally, CA-loaded environmentally friendly nanofibers developed in this study may be a promising material for novel active packaging applications requiring a high antioxidant activity and biodegradation rate. Due to their weak mechanical capabilities, it is advised that these nanofiber films with antioxidant properties be used in combination with another packaging material to make multilayered packaging.

## Figures and Tables

**Table 1:** Nanofibers’ nomenclature according to their compositions.

Name	Composition
3C1W	3% (*w*/*v*) Carob flour-1% (*w*/*v*) WPC
3C3W	3% (*w*/*v*) Carob flour-3% (*w*/*v*) WPC
3C3W1CA	3% (*w*/*v*) Carob flour-3% (*w*/*v*) WPC-1% (w/w) CA
3C3W10CA	3% (*w*/*v*) Carob flour-3% (*w*/*v*) WPC- 10% (w/w) CA

**Table 2:** Solution properties and average fiber diameters.

Solutions	n	k (Pa s^n^)	Electrical Conductivity (mS/cm)	Average Fiber Diameter (nm)
3C1W	0.9121 ± 0.0013 ^a^ *	0.386 ± 0.020 ^c^	4.03 ± 0.03 ^c^	242 ± 56 ^bc^
3C3W	0.9124 ± 0.0086 ^a^	0.545 ± 0.012 ^a^	4.10 ± 0.01 ^c^	310 ± 70 ^a^
3C3W1CA	0.9215 ± 0.0002 ^a^	0.451 ± 0.015 ^b^	4.78 ± 0.03 ^b^	257 ± 66 ^b^
3C3W10CA	0.9330 ± 0.0175 ^a^	0.267 ± 0.002 ^d^	5.69 ± 0.12 ^a^	222 ± 49 ^c^

* Columns with different letters are significantly different (*p* ≤ 0.05). k: consistency index, n: flow behavior index. r^2^ = 0.998–0.999.

**Table 3:** Glass transition temperature, melting temperature, and melting enthalpy of nanofibers.

Sample	T_g_ (°C)	T_m_ (°C)	ΔH_m_ (J· g-^1^)
3C1W	−5.28 ± 0.877 ^a^ *	63.9 ± 0.141 ^a^	40.89 ± 0.156 ^a^
3C3W	−9.36 ± 0.509 ^b^	60.92 ± 0.113 ^b^	36.47 ± 0.665 ^b^
3C3W1CA	−9.83 ± 0.467 ^b^	61.05 ± 0.071 ^b^	28.15 ± 0.071 ^c^
3C3W10CA	−9.47 ± 0.240 ^b^	62.71 ± 0.580 ^a^	28.16 ± 0.226 ^c^

* Columns with different letters are significantly different (*p* ≤ 0.05).

**Table 4:** Water vapor permeabilities of the electrospun nanofibers.

Sample	WVP×10^−10^ (g·s^−1^ ·m^−1^ ·Pa^−1^)
3C1W	2.95 ± 0.21 ^a^ *
3C3W	1.38 ± 0.14 ^c^
3C3W1CA	2.06 ± 0.08 ^b^
3C3W10CA	1.91 ± 0.10 ^bc^

* Columns with different letters are significantly different (*p* ≤ 0.05).

**Table 5:** Loading efficiency and antioxidant properties of nanofibers containing different amounts of caffeic acid and 3C3W.

Sample	LE (%)	AA (%)
3C3W	-	0.85 ± 0.03 ^c^
3C3W1CA	76.4 ± 1.3 ^b^ *	31.47 ± 0.69 ^b^
3C3W10CA	94.0 ± 1.7 ^a^	92.95 ± 1.19 ^a^

* Columns with different letters are significantly different (*p* ≤ 0.05).

## Data Availability

The data presented in this article are available on reasonable request, from the corresponding author.

## References

[1] Celebioglu A., Uyar T. (2020). Design of Polymer-Free Vitamin-A Acetate/Cyclodextrin Nanofibrous Webs: Antioxidant and Fast-Dissolving Properties. Food Funct..

[2] Vilchez A., Acevedo F., Cea M., Seeger M., Navia R. (2020). Applications of Electrospun Nanofibers with Antioxidant Properties: A Review. Nanomaterials.

[3] Rani P., Yu X., Liu H., Li K., He Y., Tian H., Kumar R. (2021). Material, Antibacterial and Anticancer Properties of Natural Polyphenols Incorporated Soy Protein Isolate: A Review. Eur. Polym. J..

[4] Guan X., Li L., Li S., Liu J., Huang K. (2020). A Food-Grade Continuous Electrospun Fiber of Hordein/Chitosan with Water Resistance. Food Biosci..

[5] Zhang C., Li Y., Wang P., Zhang H. (2020). Electrospinning of Nanofibers: Potentials and Perspectives for Active Food Packaging. Compr. Rev. Food Sci. Food Saf..

[6] Rangaraj V.M., Rambabu K., Banat F., Mittal V. (2021). Natural Antioxidants-Based Edible Active Food Packaging: An Overview of Current Advancements. Food Biosci..

[7] Shao P., Niu B., Chen H., Sun P. (2018). Fabrication and Characterization of Tea Polyphenols Loaded Pullulan-CMC Electrospun Nanofiber for Fruit Preservation. Int. J. Biol. Macromol..

[8] Alehosseini A., Gómez-mascaraque L.G., Ghorani B., López-rubio A. (2019). Stabilization of a Saffron Extract through Its Encapsulation within Electrospun/Electrosprayed Zein Structures. LWT Food Sci. Technol..

[9] Aslaner G., Sumnu G., Sahin S. (2021). Encapsulation of Grape Seed Extract in Rye Flour and Whey Protein–Based Electrospun Nanofibers. Food Bioprocess Technol..

[10] Aydogdu A., Yildiz E., Aydogdu Y., Sumnu G., Sahin S., Ayhan Z. (2019). Food Hydrocolloids Enhancing Oxidative Stability of Walnuts by Using Gallic Acid Loaded Lentil Flour Based Electrospun Nanofibers as Active Packaging Material. Food Hydrocoll..

[11] Luzi F., Torre L., Puglia D. (2020). Antioxidant Packaging Films Based on Ethylene Vinyl Alcohol Copolymer (EVOH) and Caffeic Acid. Molecules.

[12] Chen H., Wang J., Cheng Y., Wang C., Liu H., Bian H., Pan Y., Sun J., Han W. (2019). Application of Protein-Based Films and Coatings for Food Packaging: A Review. Polymers.

[13] Oguz S., Tam N., Aydogdu A., Sumnu G., Sahin S. (2018). Development of Novel Pea Flour-Based Nanofibres by Electrospinning Method. Int. J. Food Sci. Technol..

[14] Tam N., Oguz S., Aydogdu A., Sumnu G., Sahin S. (2017). Influence of Solution Properties and PH on the Fabrication of Electrospun Lentil Flour/HPMC Blend Nanofibers. Food Res. Int..

[15] Uygun E., Yildiz E., Sumnu G., Sahin S. (2020). Microwave Pretreatment for the Improvement of Physicochemical Properties of Carob Flour and Rice Starch–Based Electrospun Nanofilms. Food Bioprocess Technol..

[16] Woranuch S., Pangon A., Puagsuntia K., Subjalearndee N., Intasanta V. (2017). Rice Flour-Based Nanostructures via a Water-Based System: Transformation from Powder to Electrospun Nanofibers under Hydrogen-Bonding Induced Viscosity, Crystallinity and Improved Mechanical Property. RSC Adv..

[17] Papaefstathiou E., Agapiou A., Giannopoulos S., Kokkinofta R. (2018). Nutritional Characterization of Carobs and Traditional Carob Products. Food Sci. Nutr..

[18] Zhu B.J., Zayed M.Z., Zhu H.X., Zhao J., Li S.P. (2019). Functional Polysaccharides of Carob Fruit: A Review. Chin. Med..

[19] Muthu T.S., Kumar K.S., Rajini N., Siengchin S., Ayrilmis N., Rajulu A.V. (2019). A Comprehensive Review of Electrospun Nanofibers: Food and Packaging Perspective. Compos. Part B.

[20] Aman M., Ramazani S., Rostami M., Raeisi M., Tabibiazar M., Ghorbani M. (2019). Food Hydrocolloids Fabrication of Food-Grade Nano Fi Bers of Whey Protein Isolate—Guar Gum Using the Electrospinning Method. Food Hydrocoll..

[21] Kutzli I., Gibis M., Baier S.K., Weiss J. (2019). Food Hydrocolloids Electrospinning of Whey and Soy Protein Mixed with Maltodextrin—Influence of Protein Type and Ratio on the Production and Morphology of Fibers. Food Hydrocoll..

[22] Colín-Orozco J., Zapata-Torres M., Rodríguez-Gattorno G., Pedroza-Islas R. (2015). Properties of Poly (Ethylene Oxide)/Whey Protein Isolate Nanofibers Prepared by Electrospinning. Food Biophys..

[23] Vega-Lugo A.C., Lim L.T. (2012). Effects of Poly(Ethylene Oxide) and PH on the Electrospinning of Whey Protein Isolate. J. Polym. Sci. Part B Polym. Phys..

[24] Kuntzler S.G., Costa J.A.V., de Morais M.G. (2018). Development of Electrospun Nanofibers Containing Chitosan/PEO Blend and Phenolic Compounds with Antibacterial Activity. Int. J. Biol. Macromol..

[25] Neo Y.P., Ray S., Jin J., Gizdavic-Nikolaidis M., Nieuwoudt M.K., Liu D., Quek S.Y. (2013). Encapsulation of Food Grade Antioxidant in Natural Biopolymer by Electrospinning Technique: A Physicochemical Study Based on Zein-Gallic Acid System. Food Chem..

[26] Tatlisu N.B., Yilmaz M.T., Arici M. (2019). Fabrication and Characterization of Thymol-Loaded Nanofiber Mats as a Novel Antimould Surface Material for Coating Cheese Surface. Food Packag. Shelf Life.

[27] Yildiz E., Sumnu G., Kahyaoglu L.N. (2021). Monitoring Freshness of Chicken Breast by Using Natural Halochromic Curcumin Loaded Chitosan/PEO Nanofibers as an Intelligent Package. Int. J. Biol. Macromol..

[28] Aydogdu A., Sumnu G., Sahin S. (2019). Fabrication of Gallic Acid Loaded Hydroxypropyl Methylcellulose Nanofibers by Electrospinning Technique as Active Packaging Material. Carbohydr. Polym..

[29] Da Silva Filipini G., Romani V.P., Guimarães Martins V. (2020). Biodegradable and Active-Intelligent Films Based on Methylcellulose and Jambolão (*Syzygium cumini*) Skins Extract for Food Packaging. Food Hydrocoll..

[30] Liu Y., Liang X., Wang S., Qin W., Zhang Q. (2018). Electrospun Antimicrobial Polylactic Acid/Tea Polyphenol Nanofibers for Food-Packaging Applications. Polymers.

[31] Mohammadi M.A. (2020). Application of Electrospinning Technique in Development of Intelligent Food Packaging: A Short Review of Recent Trends. Food Sci. Nutr..

[32] Ashraf R., Sofi H.S., Malik A., Beigh M.A., Hamid R., Sheikh F.A. (2019). Recent Trends in the Fabrication of Starch Nanofibers: Electrospinning and Non-Electrospinning Routes and Their Applications in Biotechnology. Appl. Biochem. Biotechnol..

[33] Aydogdu A., Kirtil E., Sumnu G., Oztop M.H., Aydogdu Y. (2018). Utilization of Lentil Flour as a Biopolymer Source for the Development of Edible Films. J. Appl. Polym. Sci..

[34] Beikzadeh S., Akbarinejad A., Swift S., Perera J., Kilmartin P.A., Travas-Sejdic J. (2020). Cellulose Acetate Electrospun Nanofibers Encapsulating Lemon Myrtle Essential Oil as Active Agent with Potent and Sustainable Antimicrobial Activity. React. Funct. Polym..

[35] Sullivan S.T., Tang C., Kennedy A., Talwar S., Khan S.A. (2014). Food Hydrocolloids Electrospinning and Heat Treatment of Whey Protein Nano Fi Bers. Food Hydrocoll..

[36] Wilk S., Benko A. (2021). Advances in Fabricating the Electrospun Biopolymer-Based Biomaterials. J. Funct. Biomater..

[37] Zhong J., Mohan S.D., Bell A., Terry A., Mitchell G.R., Davis F.J. (2018). Electrospinning of Food-Grade Nanofibres from Whey Protein. Int. J. Biol. Macromol..

[38] Ignatova M.G., Manolova N.E., Rashkov I.B., Markova N.D., Toshkova R.A., Georgieva A.K., Nikolova E.B. (2016). Poly(3-Hydroxybutyrate)/Caffeic Acid Electrospun Fibrous Materials Coated with Polyelectrolyte Complex and Their Antibacterial Activity and In Vitro Antitumor Effect against HeLa Cells. Mater. Sci. Eng. C.

[39] Hernández-Fernández J., Rayón E., López J., Arrieta M.P. (2019). Enhancing the Thermal Stability of Polypropylene by Blending with Low Amounts of Natural Antioxidants. Macromol. Mater. Eng..

[40] Ignatova M., Manolova N., Rashkov I., Markova N. (2018). Antibacterial and Antioxidant Electrospun Materials from Poly(3-Hydroxybutyrate) and Polyvinylpyrrolidone Containing Caffeic Acid Phenethyl Ester—“In” and “on” Strategies for Enhanced Solubility. Int. J. Pharm..

[41] Nikbaht M., Salehi M., Rezayat S.M., Majidi R.F. (2020). Various Parameters in the Preparation of Chitosan/Polyethylene Oxide Electrospun Nanofibers Containing Aloe Vera Extract for Medical Applications. Nanomed. J..

[42] Yu S.H., Hsieh H.Y., Pang J.C., Tang D.W., Shih C.M., Tsai M.L., Tsai Y.C., Mi F.L. (2013). Active Films from Water-Soluble Chitosan/Cellulose Composites Incorporating Releasable Caffeic Acid for Inhibition of Lipid Oxidation in Fish Oil Emulsions. Food Hydrocoll..

[43] Benbettaieb N., Nyagaya J., Seuvre A.M., Debeaufort F. (2018). Antioxidant Activity and Release Kinetics of Caffeic and P-Coumaric Acids from Hydrocolloid-Based Active Films for Healthy Packaged Food. J. Agric. Food Chem..

[44] Araghi M., Moslehi Z., Nafchi A.M., Mostahsan A., Salamat N., Garmakhany A.D. (2015). Cold Water Fish Gelatin Modification by a Natural Phenolic Cross-Linker (Ferulic Acid and Caffeic Acid). Food Sci. Nutr..

[45] Aydogdu A., Sumnu G., Sahin S. (2018). A Novel Electrospun Hydroxypropyl Methylcellulose/Polyethylene Oxide Blend Nanofibers: Morphology and Physicochemical Properties. Carbohydr. Polym..

[46] Rodsamran P., Sothornvit R. (2019). Lime Peel Pectin Integrated with Coconut Water and Lime Peel Extract as a New Bioactive Film Sachet to Retard Soybean Oil Oxidation. Food Hydrocoll..

[47] Insaward A., Duangmal K., Mahawanich T. (2015). Mechanical, Optical, and Barrier Properties of Soy Protein Film as Affected by Phenolic Acid Addition. J. Agric. Food Chem..

[48] Fathi M., Mirlohi M., Varshosaz J., Madani G. (2013). Novel Caffeic Acid Nanocarrier: Production, Characterization, and Release Modeling. J. Nanomater..

[49] Wang A., Leible M., Lin J., Weiss J., Zhong Q. (2020). Caffeic Acid Phenethyl Ester Loaded in Skim Milk Microcapsules: Physicochemical Properties and Enhanced in Vitro Bioaccessibility and Bioactivity against Colon Cancer Cells. J. Agric. Food Chem..

[50] Chen J.H., Ho C.-T. (1997). Antioxidant Activities of Caffeic Acid and Its Related Hydroxycinnamic Acid Compounds. J. Agric. Food Chem..

[51] Youssef M.K.E., El-Manfaloty M.M., Ali H.M. (2013). Assessment of Proximate Chemical Composition, Nutritional Status, Fatty Acid Composition and Phenolic Compounds of Carob (*Ceratonia siliqua* L.). Food Public Health.

[52] Ivanković A., Talić S., Lasić M. (2017). Biodegradable packaging in the food industry. J. Food Saf. Food Qual..

[53] Alqahtani N., Alnemr T., Ali S. (2021). Development of Low-Cost Biodegradable Films from Corn Starch and Date Palm Pits (*Phoenix dactylifera*). Food Biosci..

[54] Medina-Jaramillo C., Ochoa-Yepes O., Bernal C., Famá L. (2017). Active and Smart Biodegradable Packaging Based on Starch and Natural Extracts. Carbohydr. Polym..

[55] Medina Jaramillo C., Gutiérrez T.J., Goyanes S., Bernal C., Famá L. (2016). Biodegradability and Plasticizing Effect of Yerba Mate Extract on Cassava Starch Edible Films. Carbohydr. Polym..

